# Evolution of the P-type II ATPase gene family in the fungi and presence of structural genomic changes among isolates of *Glomus intraradices*

**DOI:** 10.1186/1471-2148-6-21

**Published:** 2006-03-10

**Authors:** Nicolas Corradi, Ian R Sanders

**Affiliations:** 1 Department of Ecology and Evolution, Biology building, University of Lausanne, 1015 Lausanne, Switzerland

## Abstract

**Background:**

The P-type II ATPase gene family encodes proteins with an important role in adaptation of the cell to variation in external K^+^, Ca^2+ ^and Na^2+ ^concentrations. The presence of P-type II gene subfamilies that are specific for certain kingdoms has been reported but was sometimes contradicted by discovery of previously unknown homologous sequences in newly sequenced genomes. Members of this gene family have been sampled in all of the fungal phyla except the arbuscular mycorrhizal fungi (AMF; phylum *Glomeromycota*), which are known to play a key-role in terrestrial ecosystems and to be genetically highly variable within populations. Here we used highly degenerate primers on AMF genomic DNA to increase the sampling of fungal P-Type II ATPases and to test previous predictions about their evolution. In parallel, homologous sequences of the P-type II ATPases have been used to determine the nature and amount of polymorphism that is present at these loci among isolates of *Glomus intraradices *harvested from the same field.

**Results:**

In this study, four P-type II ATPase sub-families have been isolated from three AMF species. We show that, contrary to previous predictions, P-type IIC ATPases are present in all basal fungal taxa. Additionally, P-Type IIE ATPases should no longer be considered as exclusive to the *Ascomycota *and the *Basidiomycota*, since we also demonstrate their presence in the *Zygomycota*. Finally, a comparison of homologous sequences encoding P-type IID ATPases showed unexpectedly that indel mutations among coding regions, as well as specific gene duplications occur among AMF individuals within the same field.

**Conclusion:**

On the basis of these results we suggest that the diversification of P-Type IIC and E ATPases followed the diversification of the extant fungal phyla with independent events of gene gains and losses. Consistent with recent findings on the human genome, but at a much smaller geographic scale, we provided evidence that structural genomic changes, such as exonic indel mutations and gene duplications are less rare than previously thought and that these also occur within fungal populations.

## Background

In nature, different types of efflux/influx systems have evolved to maintain an osmotic equilibrium. These systems play a role in balancing any excessive entrance (or exit) when elevated differences in cations' concentrations are present between the surrounding and cellular environment. These systems are part of a gene super-family better known as ion pumps or plasma membrane (P-type) ATPases that evolved independently several times. Their evolution led to five main gene families (Type I, II, III, IV and V; [1]). P-type ATPases that share specificity for Ca^2+^, K^+ ^and Na^+ ^group together in a single clade and are designated as P-Type II ATPases. At present, the nomenclature of the P-Type II ATPases includes five sub-families (A, B, C, D and E; also known as SERCA, PMCA, NK/HK, ENA and ACU, respectively). These genes are known to play a key role in the adaptation of the cells to variable environments, such as variations in the PH of the soil or in cations' concentrations.

The nomenclature of the P-type II ATPase sub-family has been frequently altered in recent years. Many hypotheses concerning P-type II ATPase evolution in the fungal kingdom have been proposed by increased genome sequencing efforts and the parallel findings of new members of this gene family. The P-Type II ATPase gene family in the kingdom *Fungi *is characterized by the presence of two evolutionarily related sub-families, named P-type IIC and IIE. The latter of these is exclusively found in fungi. P-Type IIC ATPases encode ion pumps that play a role in the exchange of sodium and potassium ions across the plasma membrane, providing the energy for secondary transport of various nutrients through the creation of an electrochemical gradient [2]. These genes were thought for many years to be harboured exclusively by animal cells [3] but in the last decade several studies reported their presence in organisms that are not related to the *Metazoa*, including a basal fungal taxa (*Blastocladiella emersooni*); [3-5]. To date, these genes have been exclusively isolated from organisms that do not possess a cell wall in at least one stage of their life cycle. Accordingly, these genes were found to be absent from the *Ascomycota *and the *Basidiomycota*; two main fungal phyla where all stages of the life cycle possess a cell wall. In contrast, these two fungal phyla were shown to harbour other ATPases mediating high affinity potassium and sodium uptake. These newly identified genes were phylogenetically related to P-Type-IIC ATPases but were divergent enough from the latter sub-family to allow their description as a novel sub-family (Type IIE) [6]. Together, these results were in agreement with a prediction that P-Type IIC genes may only be found in organisms without a cell wall, at least in one stage of their life-cycle [7] and suggested a evolutionary scenario in which fungi harbouring a wall made of chitin evolved with divergent and specific potassium and sodium exchange systems, the Type IIE genes.

From analyses of fossil records and the use of molecular clocks on phylogenies it appears that the extant fungal phyla most likely diverged about one billion years ago [8-12]. Considering the long evolutionary history of these organisms, clear predictions about the evolution of their gene families should rest on comparative analyses of most of their members. The lack of knowledge concerning the presence or absence of P-Type II ATPase subfamilies in the different fungal lineages could possibly lead to wrong or simplistic predictions. Therefore, we may be far from having a conclusive view of the evolutionary history of this sub-family within the *Fungi *and any effort aimed at sampling P-Type II ATPases from yet uncovered fungal phyla is certainly warranted. At present, the sampling of P-type II genes from fungal species have only been carried out on four out of the five extant fungal phyla. P-type II ATPase gene sequences have not yet been isolated from arbuscular mycorrhizal fungi (AMF). This group of obligate symbiotic fungi has recently been erected to the status of a phylum, the *Glomeromycota *[13]. AMF are an ecologically important group of fungi, influencing plant nutrient acquisition [14], providing a protective role against plant pathogenic fungi [15] and determining plant diversity and ecosystem productivity [16]. Furthermore, they are an interesting phylum in which to study gene evolution. Firstly, although these fungi are known to be present on earth since the Devonian period [17], they show remarkably low morphological and species diversification. Secondly, they have been proposed to be multigenomic; possessing genetically different nuclei in a common cytoplasm [18,19]. Finally, little is known about the positioning of this phylum within the fungal kingdom. The most recent studies suggest that they could be a more basal phylum than previously thought [20,21]. Interestingly, although AMF are important for plant growth and have also an unusual genome organisation, sequence data about these organisms encompasses only a very few coding genes. Overall, only a small number of gene families that have arisen by gene duplication have previously been isolated from AMF species [21,22]. The isolation of, as yet, undiscovered gene families such as the P-Type II ATPase would allow us to address fundamental questions about the evolutionary history of these organisms. In particular, to shed light on features of their evolution compared to the other fungal phyla, from their positioning within the fungal clade to the understanding of molecular evolution of gene families within an entire kingdom.

Given that glomeromycotan species diversity is low, that they possess an unusual genome organisation and they are putative ancient asexuals, it would be interesting to know how important gene families such as P-Type II ATPases have evolved in this fungal phylum. To date, almost all information on sequence differences in this interesting fungal group comes from non-coding regions of the genome. Furthermore, unexpectedly high genetic variation has been recorded in a population of one AMF species, *Glomus intraradices *[23]. However, this study used markers that were probably neutral. Considerable within-AMF species genetic variability has also been recorded in genes but, again, this was restricted to introns and no selective importance could be inferred [24]. Therefore, a study of variation in P-Type II ATPases within an AMF species or population could also reveal interesting genetic variation in these putative exclusively clonal lineages.

The aims of this study were to isolate the P-type II ATPase gene family from members of the fungal phylum *Glomeromycota*. Three AMF species belonging to genus *Glomus *and cultured *in vitro *have been used here, as this culturing system allows the researcher to handle a much higher quantity and quality of DNA, which is necessary when using cloning techniques such as PCR amplifications with highly degenerate primers. In addition it greatly reduces the probability of sequencing genes from contaminant fungi, that has been a repeated technical problem with pot cultured AMF [20]. The AMF sequence data was compared with homologous sequences from other fungal phyla to determine the evolutionary history of the P-type II sub-families within the fungal kingdom. The successful amplification of the P-Type II ATPase genes from AMF also allowed us to compare their genetic variability among isolates of *Glomus intraradices *of the same population and previously reported as being highly divergent with respect to neutral markers. The results presented in this study show that the evolution of the P-Type II ATPase gene family in the fungi is more complex than previously thought. In addition, an analysis of homologous sequences sampled among isolates of *Glomus intraradices *suggested that the high genetic diversity reported using neutral markers is also present when looking at protein encoding genes of potentially high adaptive importance. Finally, we identified unexpected within population duplication events in these important genes.

## Results

### Identification of P-Type II ATPase orthologs and paralogs in AMF

**Figure 1:**
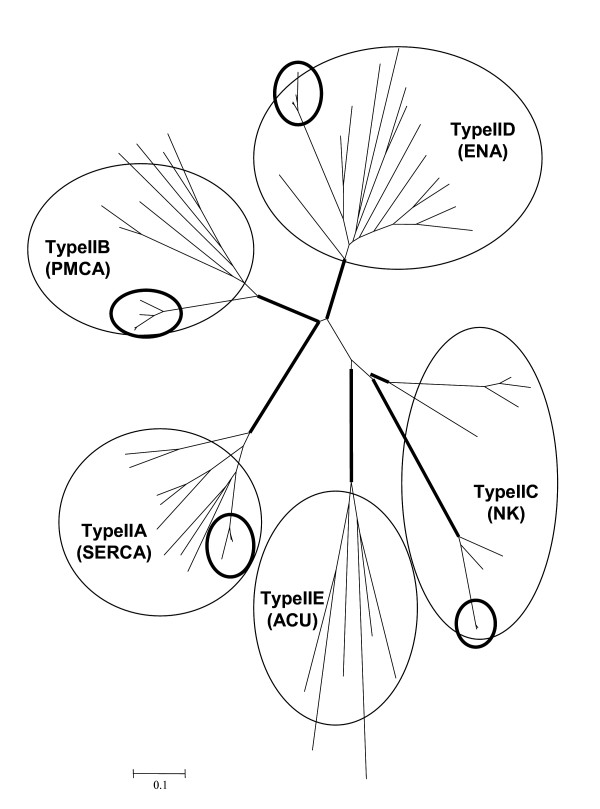
Phylogenetic analysis of P-type II amino acid sequences of various taxonomic origins. P-type II ATPase genes, obtained from arbuscular mycorrhizal fungi, were compared with previously published sequences belonging to all P-Type II sub-families. The main P-type II sub-families are circled. The placement of AMF sequences within the tree is shown by bold circles. Branches in bold had bootstrap support above 90 for both neighbour-joining and minimum evolution genetic distances. Scale bar represents 0.1 amino acid changes per site.

For all the AMF species we investigated (*Glomus intraradices *DAOM 181602; *Glomus diaphanum *and *Glomus proliferum*), the PCR with degenerate primers always yielded fragments of identical size and content when same the combinations of primers were used. Most of the sequences recovered showed highest similarities with P-Type II ATPases of fungal origin. Phylogenetic analysis of those sequences with P-Type II ATPase genes of various taxonomic origin showed that we successfully amplified AMF genes from four of the five different sub-families known to date (Fig. 1). The length, GC content and the inter-specific genetic variability found in the coding regions of these sequences are summarized in Table 3. Comparisons among coding sequences showed that not all members of the P-Type II gene family evolve similarly in AMF (Table 3). Overall, P-type IIA sequences showed less inter-specific variation (8%) compared to P-type IIC and IID ATPases (11%). P-type IIB genes showed the highest genetic variability among exons (13%) and, interestingly, this was correlated with the number of non-synonymous substitutions, which accumulated at a higher frequency in these genes. Several indel mutations were found among coding regions for both orthologs and paralogs of AMF sequences we recovered. Indel mutations were shown to vary in length from a minimum of six base pairs among P-Type IIA sequences to a maximum of 24 base pairs as identified among paralogs of P-Type IIB ATPases. Indel mutations neither led to a shift in the open reading frame (ORF), nor to the appearance of termination codons in the coding regions.

**Table 3:** Number of isoforms and variability in coding regions among P-Type-II ATPases isolated from *Glomus *spp. *The GC content has been averaged when paralogs have been identified.

Type-II ATPase	*Glomus *spp.	Length of the anaysed fragment	GC content*	# isoforms	% variability among species	% variability between paralogs	polymorphic indels among species (paralogs)
**Type-IIA (SERCA)**							
	*Glomus intraradices *(DAOM 181602)	1933 bp	37%	1	-	-	
	*Glomus diaphanum*	1141 bp	37%	1	-	-	
	*Glomus proliferum*	1141 bp	38%	1	-	-	
	overall				8%		6 bp
**Type-IIB (PMCA)**							
	*Glomus intraradices *(DAOM 181602)	1013 and 1037 bp	37.5%	2	-	5%	(24 bp)
	*Glomus diaphanum*	1013 bp	37%	1	-	-	-
	*Glomus proliferum*	1013 bp	38%	1	-	-	-
	overall				13%		24 bp
**Type-IIC (NaK)**							
	*Glomus intraradices *(DAOM 181602)	917 bp	33%	2	-	5%	-
	*Glomus diaphanum*	917 bp	31%	2	-	4%	-
	*Glomus proliferum*	917 bp	32%	1	-	-	-
	overall				11%		
**Type-IID (ENA)**							
	*Glomus intraradices *(DAOM 181602)	2589 and 2401	34.5%	2	-	3%	(9 bp, 6 bp)
	*Glomus diaphanum*	850 bp	38%	2	-	3%	-
	*Glomus proliferum*	856 bp	38.5%	1	-	-	-
	overall				11%		9 bp, 6 bp, 6 bp

**Figure 2:**
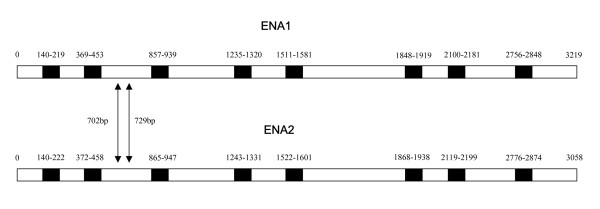
Representation, at the nucleotide level, of the complete open reading frame of the two paralogs encoding P-Type IID ATPases and recovered from *Glomus intraradices *(DAOM 181602). The positions of the introns along the paralogs are marked in black. The positions of two indel mutations along the paralogs are indicated by arrows.

Through a first-strand cDNA synthesis from mRNA of *Glomus intraradices *(DAOM 181602) and successive cloning procedures, we were able to identify the coding regions of all the sequences we isolated from genomic AMF DNA. This included identification of gene duplicates. This is direct evidence that all members of this gene family are constitutively expressed under the standard conditions of *in vitro *culturing. Of the gene family analysed in this study, only the Type IID sub-family was expressed in sufficient amounts to allow the isolation of the complete ORF through the RACE procedure. Sequence analysis of the entire coding region of the P-type IID ATPase gene showed that both gene duplicates share the same number of introns and differ by several substitutions in their coding regions, as well as the presence of two indel mutations (Fig. 2). Gene duplicates were named *ENA1 *and *ENA2 *and encode proteins of 87.7 and 87.9 kDa, respectively, with eight predicted trans-membrane domains.

**Figure 3:**
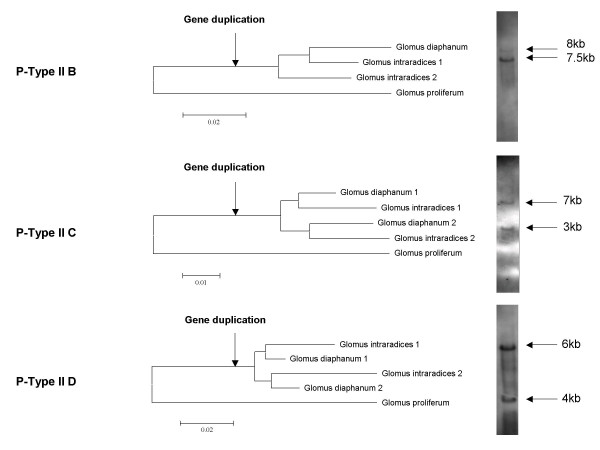
Phylogenetic analyses of synonymous substitutions among glomeromycotan P-Type IIB, C and D ATPases. The paralogous sequences were numbered according to their clustering in the phylogeny. Scale bar represents *n *substitutions per site depending on the gene. Southern blotting of the genes in *Glomus intraradices *when genomic DNA was digested using EcoRI is shown next to each phylogenetic analysis, respectively.

For the P-Type IIC and D ATPases, several variants of the same gene were isolated from *Glomus intraradices *and *Glomus diaphanum*. In contrast, no intra-individual variation was found in *Glomus proliferum*. For the P-type IIB subfamily, presence of gene duplicates was detected only in *Glomus intraradices *(DAOM 181602) (Table 3). A phylogenetic analysis of gene variants based on synonymous substitutions showed that all gene duplications identified in this study have arisen relatively recently in AMF evolution (Fig. 3). Indeed, the duplication events took place after the divergence of *Glomus intraradices *and *Glomus diaphanum *from a common ancestor with *Glomus proliferum*. Southern blotting confirmed the duplicative status of Type II B, C and D ATPases in *Glomus intraradices*, showing a banding pattern consistent with the number of variants we identified when a frequent cutting enzyme (EcoRI) was used to digest genomic DNA. When EcoRV and XbaI were used to digest genomic DNA the blotting always resulted in a single band for all the genes we analysed (data not shown).

### Evolution of fungal P-Type II ATPase sub-families

**Figure 4:**
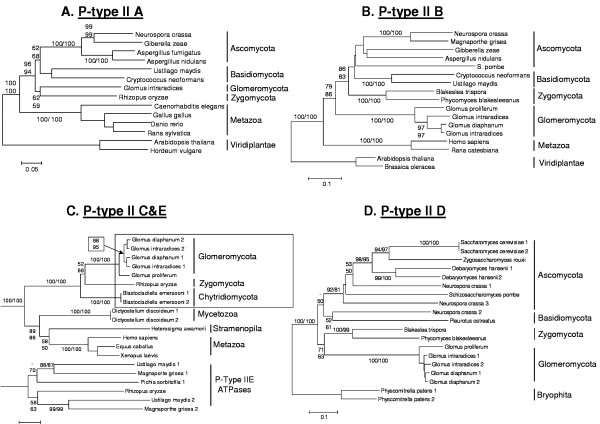
Phylogenetic analyses of the P-type II sub-families recovered using amino acid sequences of various taxonimic origin. A. Phylogeny based on P-type IIA gene sequences. B. Phylogeny based on P-type IIB gene sequences. C. Phylogeny based on P-type IIC and E gene sequences. The box indicates the fungal specific lineage of P-type IIC ATPases. D. Phylogeny based on P-type IID gene sequences. P-type II ATPase genes obtained from glomeromycotan species were compared with previously published sequences belonging to the same sub-family. Numbers at nodes represent bootstrap support for neighbour-joining (top, left) and minimum evolution (bottom, right) genetic distances. Scale bar represents *n *amino acid changes per site.

Phylogenetic analyses of P-type II ATPases were carried out to detect the placement of the Phylum *Glomeromycota *in comparison with the other fungal phyla (*Ascomycota*, *Basidiomycota, Chytridiomycota *and *Zygomycota*), as well as comparing molecular evolution among P-type II gene sub-families (Fig 4). Independently from the genes we analysed, the phylogenies always revealed a strongly supported clade, including only sequences of fungal origin. Accordingly, the glomeromycotan sequences were shown to be of fungal origin and to be monophyletic, including all the paralogs. Moreover, the clustering among AMF species used in this study was consistent among phylogenies and with other studies [20,21], thus rejecting the possibility of contaminant non-AMF sequences within our dataset.

Within the fungal clade, variation in the positioning of the fungal phyla was found according to which genes we analysed. When sequences of the *Ascomycota *and the *Basidiomycota *were available for phylogenetic analysis (Fig 4A,B and 4D), their currently accepted evolutionary relationship was found [25-27], though only the P-type IIA genes led to a support that was relatively high (Fig 4A). For the P-Type IID gene sequences, the clustering of a *Neurospora crassa *sequence with a member of the *Basidiomycota *has been already documented [28]. The phylogenies based on P-type II A and D resulted in AMF having higher similarities with members of the *Zygomycota*. In contrast, the phylogeny inferred with the P-type IIB sub-family members placed the AMF species at a basal position in the fungal clade.

The phylogeny of P-Type IIC ATPases also includes P-Type IIE ATPases as both have been reported as being phylogenetically related [6]. As expected, the P-type II sub-families formed highly supported clades. Two sequences we identified in the *Rhizopus oryzae *genome database were evolutionarily divergent and clustered in both of the main clades, providing first evidence that both P-Type IIC and E ATPases evolved in members of the *Zygomycota*. The sequences we identified from AMF showed highest sequence similarities with the Type IIC ATPases and clustered with one of the sequences from *Rhizopus oryzae*. P-type IIC ATPases of fungal origin grouped together with high bootstrap support, leading to a lineage that evolved independently from animal-like P-type IIC ATPases.

### Genetic variability and relative copy number of type IID ATPases among isolates of *Glomus intraradices*

In this study, P-Type II ATPase genes were also identified in two additional isolates of *Glomus intraradices *(*C2 *and *C3*) that were harvested from the same field and shown to be highly divergent using neutral markers [23]. We were, therefore, able to detect genetic variability that is present in adaptively important genes among isolates of the same AMF species. Sequencing efforts showed that P-Type IIA, B and C ATPases harbour a small number of nucleotide polymorphisms among these isolates and, overall, only single substitutions have been recovered among their coding regions and introns. Additionally, the number of gene duplicates we recovered was always consistent among the isolates for these three gene sub-families (data not shown).

**Figure 5:**
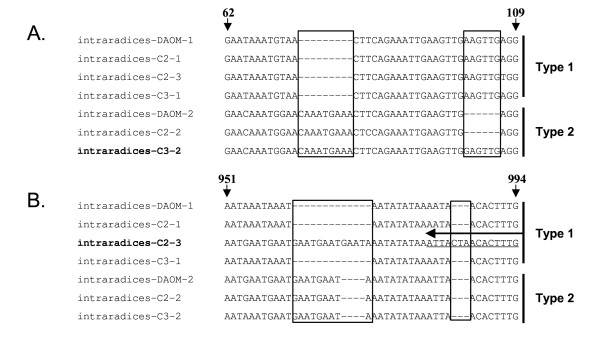
Partial nucleotide sequences of the P-Type IID ATPases isolated from three isolates of *Glomus intraradices *(DAOM 181602, C2 and C3). A. Alignment showing variation in indel mutations (in boxes) among exons of *Glomus intraradices *isolates. B. Alignment showing specific indel mutations (in boxes) lying in an intron of the additional paralog (C2-3) identified in the isolate C2. The positions of the alignments along the open reading frame are indicated by vertical arrows. Horizontal arrows indicate the 3'- annealing site that allows specific amplification of variant C2-3. Sequences specific to a single isolate are named in bold.

In contrast, the level of genetic variability was much higher among coding regions and introns of the P-Type IID ATPases of the *Glomus intraradices *isolates (Fig 5A and 5B). Indeed, not only did these genes show the presence of isolate-specific substitutions among exons and introns, in addition, indel mutations among these regions were also present. Two gene duplicates were recovered from the isolate *C3 *and these were found as phylogenetically related to those recovered from the isolate DAOM 181602. This suggests an origin through the same duplicative event, which preceded the divergence of *Glomus intraradices *and *Glomus diaphanum*. However, but one of these two paralogs (named C3-2) differed by the absence of an indel mutation in its coding region (Fig 5A). In addition to the presence of variable indel mutations among exons, another isolate, named *C2 *and which originated from the same field as *C3*, was unexpectedly found to harbour three different genes encoding a P-Type IID ATPase. Two of the variants were similar to the ones recovered from the isolate DAOM 181602, but one (named C2-3) differed from the others by the presence of two specific indel mutations within one of its introns (Fig. 5B). This additional gene variant was exclusively found in the isolate *C3 *through a PCR survey using a reverse primer partially overlapping one of its specific indel mutations (Fig. 5B).

**Figure 6:**
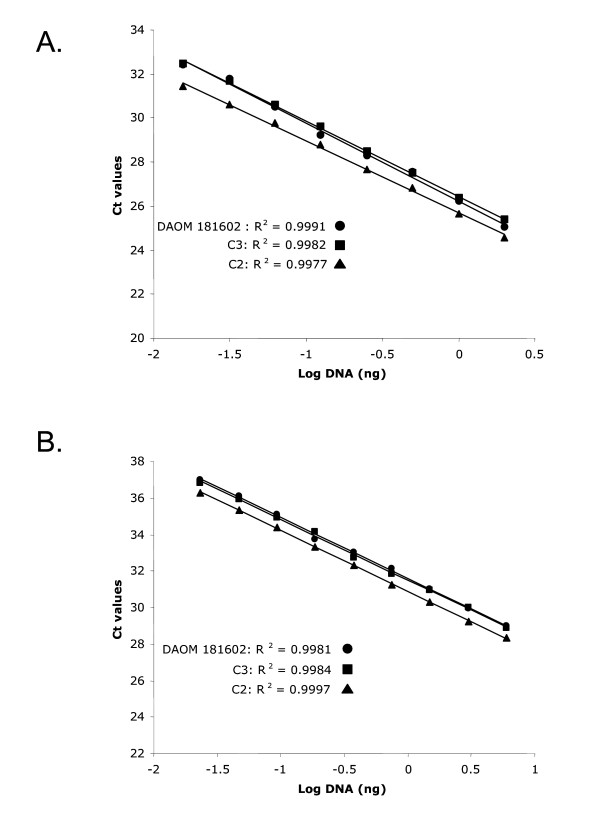
Linear regressions relating the cycle threshold parameter values (Ct values) and the log concentration of genomic DNA of isolates DAOM 181602, C2 and C3. A. Primer set and probe 1. B. Primer set and probe 2.

The amplification and cloning approach provided a first line of evidence suggesting that the number of genes encoding Type IID ATPases may differ in their number among isolates of *Glomus intraradices*. Although it would be unlikely that we missed an additional variant in the isolates DAOM 181602 and *C3 *by using highly degenerate primers as well providing an in-deep sequencing effort, the absence of a third unidentified variant in the isolates DAOM 181602 and *C3 *could not be ruled out conclusively. To test whether the isolates of *Glomus intraradices *shared the same copy number of these genes, we performed a relative quantification of the different genes using two sets of primers and probes, as well as using material originating from two independent DNA extractions of all isolates (Fig 6A and 6B). The two sets of primers and probes resulted in very similar results regarding the relative copy number of the target genes and showed that, for a given amount of genomic DNA, the isolate *C2 *always harboured a larger relative number of copies for the genes encoding a P-Type IID ATPase. The difference in the relative number of the target gene was reproducible with respect to the primer combinations and the independent DNA extraction. On average, the isolate *C2 *harboured approximately 48.5% more copies (StDev = 5.7) of P-Type IID ATPase genes compared to the isolates DAOM 181602 and *C3 *for the same amount of genomic DNA. In contrast, isolates DAOM 181602 and *C3 *did not show any significant difference in their relative copy number and, therefore, it appears that both possess a same number of genes encoding P-Type IID ATPases.

## Discussion

### P-Type II C and E ATPases evolved in basal fungal phyla

Previous studies aimed at isolating and phylogenetically analysing P-type II ATPase genes showed that the P-type IIC sub-family was only found in organisms without a cell wall, in at least one stage of their life cycle. This was consistent with the finding of a P-Type IIC ATPase in the chytrid fungus *Blastocladiella emersooni *[7]. In parallel the *Ascomycota *and the *Basidiomycota*, fungal phyla that harbour a chitinous cell wall, were found to possess an independent but phylogenetically related sub-family named P-type IIE [6]. The data were, therefore, consistent with a simplistic prediction that a lineage of the P-Type II ATPases evolved in fungi with a chitinous cell wall that could fulfil similar osmotic challenges. Our study provided evidence for more complex trends in the evolution of the P-Type II ATPase gene family in *Fungi *than previously thought. Sequences phylogenetically related to the Type IIC sub-family have been isolated from species of the *Glomeromycota *and identified in the *Rhizopus oryzae *(*Zygomycota*) genome database [29]. Within the P-Type IIC clade, those sequences were related to genes isolated from *Blastocladiella emersooni *(*Chytridiomycota*) and were shown to form a strongly supported monophyletic group. Interestingly, this cluster was shown to evolve independently from members of the same sub-family and most likely correspond to a fungal specific lineage that arose early in fungal evolution and evolved in all basal fungal phyla. As a consequence, our data do not support the hypothesis that these genes have arisen to fulfil the requests of an organism lacking a cell wall. Indeed, the zygomycete *Rhizopus oryzae *possesses a cell wall in all known stages of its life cycle and this is also the case for the *Glomeromycota*. Additionally, according to a survey of nineteen completely sequenced genomes [29], the *Ascomycota *and the *Basidiomycota *do not harbour these genes. Our data on the presence of P-type IIC *ATPases *in the basal fungal phyla (*Chytridiomycota*, *Glomeromycota *and *Zygomycota*), along with their absence in the *Ascomycota *and the *Basidiomycota*, indicates that these genes have been lost in lineages leading to higher fungi.

In addition to the absence of P-Type IIC in the *Ascomycota *and the *Basidiomycota*, our results show that the P-Type IIE ATPases can no longer be considered as exclusive to the *Ascomycota *and the *Basidiomycota*. Therefore, the presence and evolution of the Type IIE ATPases in fungi can neither be easily explained by the presence of a cell wall in a fungal organism nor by an exclusive evolution in the ascomycotan and basidiomycotan clade. We did not find any evidence for the presence of such genes in the *Chytridiomycota *and the *Glomeromycota*. Even though several combinations of primers could potentially anneal to conserved regions of fungal P-Type IIE ATPases, none of these led to the amplification of an glomeromycotan sequence showing such homologies. Two predictions can be inferred from the absence of a positive amplification of these genes in the AMF species we studied: 1. Type IIE sub-family is absent in glomeromycotan genomes or 2. Members of this sub-family underwent evolution at conserved amino acid motifs, thus impeding the amplification with degenerate primers. At present, the two predictions cannot be confirmed but data from genome sequencing projects on AMF [29] and chytrids will help in answering this question.

### Structural genomic changes in a population of *Glomus intraradices*

Indel mutations among coding regions and gene duplications have both been considered for many years as being rare evolutionary events [31] sometimes leading to the complete reappraisal of important phylogenetic questions that previously relied on nucleotide substitutions patterns [32]. In our study we showed that both of these supposedly rare events occur among AMF individuals of the same species (*Glomus intraradices*), and even from the same population. Indeed, the isolate C3 was shown to harbour two paralogous sequences encoding P-Type IID ATPases, one of which differed from the paralogs isolated in DAOM 181602 by the absence of an indel mutation in the coding region. To date, the presence of indel mutations among AMF isolates was only reported for introns [24], and no studies reported intra-population variation of this nature in coding regions of genes with potential high ecological importance. As a consequence, the extensive intra-isolate genetic variability previously found with assumed neutral markers [23] does not only involve single substitutions, but also rare genomic changes that can potentially affect the function of genes.

In addition, our data showed that gene duplication events occurred independently among isolates of *Glomus intraradices *from the same population. Lines of evidence coming from cloning and sequencing, targeted specific PCR, Southern blotting and quantification of relative differences in copy number among isolates of *Glomus intraradices *strongly suggest that an additional duplicative event of the P-type IID ATPase occurred independently in the isolate *C2*. The isolate *C2 *was found to harbour relatively fifty percent more copies compared to other isolates. This is exactly what we would expect if the isolate *C2 *would harbour three copies of the gene due to additional gene duplication. The finding that *Glomus intraradices *individuals do not share the same copy number for genes of potentially ecological importance is interesting, suggesting a possible variation in the adaptation of these organisms to variable environments and could explain, in part, the intra-specific phenotypic variability recorded by others [23].

## Conclusion

The results presented in this study about fungal P-Type II ATPases show that predictions about the evolution of gene families should rely in the future on analyses including a broad sampling of organisms for a given phylum. Most of the gene families presumably originated early in the evolution of eukaryotes and prokaryotes and followed several events of gene gains or losses along distinct evolutionary lineages. Consequently, the analyses of gene families should require a broad sampling of taxa to avoid biases in the phylogenetic results and simplistic predictions about their evolution. The addition of previously unknown fungal P-type II ATPase gene sequences brought new interesting insights about the evolution of the gene family as a whole and showed the presence of new evolutionary sub-clades.

The obligate symbiotic status of AMF and the evolutionary processes we described in this study open exciting perspectives about the analysis of the P-type II ATPase gene expression in fungal organisms and especially in AMF. By isolating the putative entire family of P-Type II ATPases from AMF species, we provided sequence data of use for future studies in this direction.

Finally, it has to be noted that the genomic events we reported as occurring among isolates of *Glomus intraradices *have been also recently reported as occurring among individuals in humans [33,34] and, recently, have been shown to be of high selective importance against diseases [35,36]. Additionally, in the literature, we found other evidence for a variation in gene copy number among isolates of *Glomus intraradices*, although, this was related to ribosomal genes (25S rDNA) and not specifically discussed by the authors (Fig. 4a in [37]). In conclusion, it appears that our results confirm the expectations that genomic changes previously thought to occur rarely in the evolution of organisms can be rather frequent. Indeed, these events do not only appear among highly divergent lineages but can also occur among highly related species and individuals from the same population.

## Methods

### AMF cultivation, genomic DNA and RNA extraction

Isolates of *Glomus intraradices *DAOM 181602; *C2 *and *C3*, (the latter two harvested from the same field in Switzerland and named according to [23]), *Glomus diaphanum *(MUCL 43196), and *Glomus proliferum *(MUCL 41827) were grown with Ri T-DNA transformed *Daucus carrota *roots. These fungi were maintained on two-compartment plates that allowed proliferation of large amounts of hyphae and spores in a compartment that is free of roots [38]. Roots that directed their growth to the fungal compartment were regularly cut to avoid contamination with plant DNA. The cultures were grown for an average period of 3 months at 25°C before DNA extraction. Spores and hyphae were harvested by dissolving the medium in citric acid [39]. Fungal material was collected on a 22 μm sieve and used for DNA extraction. Freshly harvested mycelium was placed in a 2 ml microcentrifuge tube and disrupted using a magnetic stirrer (Cenco instruments) and a micro-stirring bar for 10 min at 1200 rpm. DNA was then extracted from the resulting suspensions using the DNeasy plant mini kit (Qiagen). DNA concentration was estimated using a fluorimeter (Hoefer DyNA Quant 200) and DNA quality was checked by migration of 150 ng DNA on a 0.6 % TAE agarose gel after electrophoretic separation for 30 minutes at 8 V per cm. RNA was extracted from freshly harvested mycelium by using the *ToTALLY RNA*™ Kit (Ambion, UK) according to manufacturer's protocol.

### Gene amplification and DNA cloning

A total of eight different degenerate primers were designed for conserved regions of P-type II ATPases and are listed in Table 1. Amplifications were carried out in a final volume of 50 μl containing 1× PCR buffer (10 mM Tris-HCl, 50 mM KCl, 1.5 mM MgCl2, 0.1% Triton X 100, 0.2 mg/ml BSA), 100 nM dNTP, 1 μM final concentration of each degenerate primer, and 0.5 units of *Accu-prime*™ polymerase (Invitrogen, Inc.). PCR was performed in an automated thermal cycler (T-gradient, Biometra) with an initial denaturation step of 3 min at 94°C, followed by 35 cycles of denaturation for 30 s at 94°C, annealing for 45 s at 44°C and extension for 2 min at 72°C, followed by a final extension for 10 min at 72°C.

**Table 1:** List of degenerate primers and primers used for the reverse transcription PCR (RT-PCR), rapid amplification of cDNA ends (RACE) and Real-time PCR. The black vertical bar next to the primer names indicates which combinations of primers were used together.

**Degenerate primers**	**sense**	**5'-3' sequence**
ATP.Deg1	Forward	TGY WSY GAY AAR ACY GGI AC
ATP.Deg2	Reverse	TTV ACH CCR TCH CCI GTC AT
ATP.Deg3	Reverse	ATN SWN GCR AAR TTR TCR TC
ATP.Deg4	Forward	TGY WSN GAY AAK ACN GG
ATP.Deg5	Reverse	ACN CCR TCN CCN GTC AT
ATP.deg6	Forward	NGC RAA RTT RTC RTC CAT
ATP.Deg7	Reverse	AAR TTR TCR TCC ATN ARD AT
ATP.Deg8	Reverse	TAR RTN RTN CCN GCN GGN

**RT-PCR**	**sense**	**5'-3' sequence**

SERCAGlo.F	Forward	ACA ACG AAC CGT ATG AGC GT
SERCAGlo.R	Reverse	GCA AGA ACC ATA TCA GCA GC
PMCAGlo.F	Forward	ACC CAA AAC AGA ATG ACC
PMCAGlo.R	Reverse	ATC GAA GAA GCT TCC TTA GC
NaKGlo.F	Forward	TTA ACT CGA AAT CAA ATG AC
NaKGlo.R	Reverse	CGT GCA AAA ATA ATT TCA TC
ENAGlo.F	Forward	ACA CTA ACA CAG AGT AAA ATG
ENAGlo.R	Reverse	AAT CAT TTT TAC TTT TGT ATC

**RACE**	**sense**	**5'-3' sequence**

SERCA.GSP1	5' RACE	CGC TCA TAC GGT TCG TTG TAA GCG T
SERCA.GSP2	3' RACE	TAT AGC AAT GGG TGA CGG TAC CGA T
PMCA.GSP1	5' RACE	CAC CAG TAG CAG GTG CTT TTC CGT G
PMCA.GSP2	3' RACE	CTT GCT CGT TCT TCT CCA ACC GAT A
NK.GSP1	5' RACE	AGC ATC ACC AAG AAT TGC ACG TTC A
NK.GSP2	3' RACE	ACT GGT CGA CCT ATT GAG TCA ATT C
ENA.GSP1	5' RACE	GAA GCC AAG CAT CAG TTG CAA TCA T
ENA.GSP2	3' RACE	TGA CTG CCG CAC AGT TTG ATG CAC T

**Real-time PCR**	**sense**	**5'-3' sequence**

ENA.real.F1	Forward	AAC TTG CAA GCA AAG GGA TG
ENA.probe 1	Probe	TTG GCA GCA TAC CGT CGA GTT
ENA.real.R1	Reverse	GTG GAT CAT AAA TAC CAA CCA
ENA.real.F2	Forward	CAA GAA TAT GCG TTT GAC ACT GAA
ENA.probe 2	Probe	TGA AAC GTA TGT CTG TTG TAT GTA AAG AAA AAT CTA CGG A
ENA.real.R2	Reverse	AAA CAG ATT CTG TTG CGC CTT TA

In order to detect coding regions of AMF P-Type II ATPases, cDNA was synthesized by reverse transcription using the *RevertAid*™ Kit (MBI Fermentas) following the manufacturer's instructions. One microliter of the resulting cDNA was amplified in a PCR using primer combinations listed in Table 1. The amplification was performed in a final volume of 25 μl containing 1× PCR buffer (10 mM Tris-HCl, 50 mM KCl, 1.5 mM MgCl2, 0.1% Triton X 100, 0.2 mg/ml BSA), 100 nM dNTP, 0.5 μM final concentration of each primer, and 0.25 units of *Accu-prime*™ polymerase (Invitrogen, Inc.), in an automated thermal cycler (T-gradient, Biometra) with an initial denaturation step of 3 min at 94°C, followed by 30 cycles of denaturation for 30 s at 94°C, annealing for 30 s at 58°C and extension for 2 min at 72°C, followed by a final extension for 7 min at 72°C. To obtain full-length cDNA fragments of P-Type II ATPase genes, the partial cDNA fragments were lengthened by the 5'- and 3'-rapid amplification of cDNA ends (RACE) method by using the SMART-RACE™ Kit (Clontech) with the appropriate primers listed in Table 1. RACE was only performed with total RNA isolated from *Glomus intraradices *(isolate DAOM 181602) cultured *in vitro*.

Amplification products were electrophoretically separated on 1.2 % agarose gels, stained with ethidium bromide and the expected bands were excised from the gel and then purified with a Qiaquick gel extraction kit (Qiagen, GmBH). PCR fragments were then cloned into the pTZ57R/T vector (Mbi Fermentas GMBH), following the manufacturer's instructions. Sequences were obtained using M13 forward and reverse primers with the Big Dye 3.1 Terminator cycle sequencing kit (Applied Biosystems), according to the manufacturer's instructions, and separated on an ABI Prism 3100 genetic analyser (Applied Biosystems).

### Sequence analysis

Sequences were analysed using the Vector NTI package (Informax. Inc, Oxford, U.K) and homology with genes deposited in databases was conducted using BLAST [40]. Sequences were aligned using clustal W [41] and refined by eye. Phylogenetic tree reconstruction based on amino acid sequences was carried out using MEGA2 [42] and a Poisson correction for multiple substitutions at a site. Events of gene duplications between AMF genes were assessed using a tree topology recovered from synonymous substitutions and with a K2P model, including only sequences of the same sub-family and recovered from AMF species used in this study. Neighbour-joining and minimum evolution [43] genetic distances were calculated using MEGA2 for both nucleotide and amino acid sequence data. For a phylogenetic analysis of the P-type IIA gene sub-family, sequences of 381 amino acids were available from the *Zygomycota *in gene databases. However, the resulting phylogeny did not support fungal sequences as a natural monophyletic group (below 50% bootstrap values). We, therefore, increased the phylogenetically informative data by recovering a longer zygomycotan gene sequence from the *Rhizopus oryzae *genome database [29] and by performing a 3'-RACE with cDNA from *Glomus intraradices *(isolate DAOM 181602). The results allowed us to infer a phylogeny based on 568 amino acids, which strongly supported the *Fungi *as monophyletic. Consequently, the resulting phylogeny only includes a single sequence from the *Glomeromycota *and the *Zygomycota*. All phylogenetic tree topologies were assessed by 1000 bootstrap replicates. Sequences belonging to Type-IID ATPases have been found to be absent from the *Ascomycota *and the *Basidiomycota *through a survey of nineteen completely sequenced genomes [29] and, therefore, these two phyla are absent from the phylogeny recovered using these genes. Accession numbers of amino acid sequences and the list of taxa used in the phylogenetic analyses are provided in Table 2.

**Table 2:** Loci, taxa and accession number of amino acid sequences used in this study. Sequences marked with * have been recovered from the *Rhizopus oryzae *Sequencing Project (Broad Institute of Harvard and MIT; [29]).

**Locus**	**Taxon names**	**Accession numbers**
**Type IIA (SERCA ATPase)**	**Fungi**	
	*Zygomycota*	
	*Rhizopus oryzae*	super-contig 1.52*
	*Ascomycota*	
	*Neurospora crassa*	CAB65295
	*Magnaporthe grisea*	EAA50791
	*Aspergillus fumigatus*	EAL88529
	*Aspergillus nidulans*	EAA62836
	*Giberella zeae*	EAA70574
	*Basidiomycota*	
	*Ustilago maydis*	CAE11789
	*Cryptococcus neoformans*	EAL17724
	**Metazoa**	
	*Caenorhabditis elegans*	NP399385
	*Danio rerio*	CAE50627
	*Rana sylvatica*	CAC20903
	*Gallus gallus*	CAB38029
	**Viridiplantae**	
	*Arabidopsis thaliana*	AAF75073
	*Hordeum vulgare*	CAC40034

**Type IIB (PMCA ATPase)**	**Fungi**	
	*Zygomycota*	
	*Blakeslea trispora*	CAD12644
	*Phycomyces blakesleeanus*	CAD12642
	*Ascomycota*	
	*Schizosaccharomyces pombe*	CAC21470
	*Neurospora crassa*	CAD70559
	*Gibberella zeae*	EAA75993
	*Magnaporthe grisea*	EAA52198
	*Aspergillus nidulans*	EAA66307
	*Basidiomycota*	
	*Cryptococcus neoformans*	EAL21227
	*Ustilago maydis*	EAK88608
	**Metazoa**	
	*Homo sapiens*	CAD97686
	*Rana catesbiana*	AAk11273
	**Viridiplantae**	
	*Arabidopsis thaliana*	AAB84338
	*Brassica oleracea*	CAA68234

**Type IIC (NK ATPase)**	**Fungi**	
	*Zygomycota*	
	*Rhizopus oryzae*	supercontig 1.53*
	*Chytridiomycota*	
	*Blastocladiella emersonii *NK1	AAF20202
	*Blastocladiella emersonii *NK2	CAA04499
	**Metazoa**	
	*Equus caballus*	CAA34716
	*Xenopus leavis*	AAH43743
	*Homo sapiens*	AAQ07964
	**Stramenopiles**	
	*Heterosigma awamorii*	BAA82752
	**Mycetozoa**	
	*Dictyostelium discoideum*	AAO51258

**Type IID (ENA ATPase)**	**Fungi**	
	*Zygomycota*	
	*Blakeslea trispora*	CAD12643
	*Phycomyces blakesleeanus*	CAD12641
	*Ascomycota*	
	*Neurospora crassa *ENA1	CAB65298
	*Neurospora crassa *ENA2	CAB65297
	*Neurospora crassa *ENA1	XP328672
	*Debaryomyces hansenii *ENA1	AAB86427
	*Debaryomyces hansenii *ENA2	CAG85830
	*Saccharomyces cerevisiae *ENA1	NP010325
	*Saccharomyces cerevisiae *ENA2	CAA98866
	*Zygosaccharomyces rouxii*	T43270
	*Schizosaccharomyces pombe*	CAB46699
	*Basidiomycota*	
	*Pleurotus ostreatus*	CAD12640
	**Briophyta**	
	*Physcomitrella patens *ENA1	CAD91917
	*Physcomitrella patens *ENA2	CAD91919

A total of twenty-two glomeromycotan sequences, including two complete open reading frames (ORFs), were newly identified in this study and deposited in public databases under accession numbers AM118102 to AM118123.

### Southern blot analysis

Southern blot analyses were performed in order to look at most probable copy number of the P-Type II ATPases in *Glomus intraradices *(DAOM 181602). Three μg of genomic DNA extracted from *Glomus intraradices *(isolate DAOM 181602) were digested with each of the endonucleases EcoRI, EcoRV and XbaI. The samples were fractionated on a 1% agarose gel, and blotted onto a positively charged nylon membrane (Roche, Mannheim, Germany) according to standard procedures. The membranes hybridised overnight with digoxigenin (DIG)-labelled probes in an appropriate hybridisation buffer (DIG Easy Hyb, Roche) at 40°C. After hybridisation, the blot was washed twice in 2× SSC/0.1% SDS at room temperature for 5 min and twice in 0.5× SSC/0.1% SDS at 65°C for 15 min each wash. Signals on the blot were detected by the chemi-luminescent method using DIG Luminescent Detection Kit (Roche) and exposed to X-ray film. The probes corresponding to P-Type-IIB, C and D ATPases were obtained by PCR amplification using genomic DNA as template with the same specific primers used in the reverse transcription PCR (Table 1).

### Real-time PCR procedures

Real-time PCR was performed on the three *Glomus intraradices *isolates to compare relative copy number of the P-Type IIC ATPases among isolates. From the P-Type IID ATPase sequences we obtained from the different *Glomus intraradices *isolates (DAOM 181602, *C2 *and *C3*), we designed two sets of primers that amplify a 200 bp and a 70 bp region fragment, respectively, and that annealed to a region conserved among the variants we recovered. The primer combinations and probes are listed in Table 1. In a standard PCR, these primers were shown to amplify the expected gene variants from the *Glomus intraradices *isolates. These probes were labelled with FAM at the 5' end and TAMRA at the 3' end. In the real-time PCR reaction, the fluorescence of the probe was measured at each cycle at the annealing phase of the reaction. FAM-real-time PCR amplification was performed in 40 ml containing 1X qPCR Mastermix (Eurogentec), 0.5 μM each primer, 0.25 μM probe, and 10 μl DNA at different concentrations. The thermal cycling conditions were an initial step of 50°C for 2 min and 95°C for 10 min, followed by 45 cycles of 95°C for 15 s and 60°C for 1 min. Fluorescence data were collected using the ABI PRISM 7000 Sequence Detection System (SDS; Applied Biosystems). The SDS software then generated each real-time PCR profile after multi-component analysis by plotting the log of the change in fluorescence (delta Rn) versus cycle number. The cycle threshold (Ct) was determined by the SDS software as the fractional cycle number. This indicates where the fluorescence crosses an arbitrary threshold intersecting the signal curves in their exponential phases. In each experiment, two-fold serial dilutions of *Glomus intraradices *genomic DNA from the different isolates (ranging between 2000 and 16.5 pg) were included to generate Ct values. Four independent experiments were performed, each with eight concentrations of the genomic DNA. In half of the experiments, the DNA of the *Glomus intraradices *isolates was obtained from two independent extractions, thus allowing the possibility of checking whether a bias occurred in the Ct values because of DNA extraction procedures. All replicates offered very similar results and produced standard curves with regression coefficients (*R*^2^) > 0.99. The relative copy number of P-type IID genes in each of the *Glomus intraradices *isolates could then be calculated easily, by comparing the Ct values of each of the isolates for a given amount of genomic DNA. Similar Ct values for the same amount of genomic DNA would suggest the same copy number of the target between the isolates and, in contrast, differences in Ct values for a given amount of DNA would suggest differences in the number of the target per genome in the different isolates of *Glomus intraradices*. The slopes resulting from the analyses of Ct values respective to the Log of the amount of genomic DNA were consistent and reproducible among isolates and the efficiency of the quantitative PCR reaction from genomic DNA was found to be more than 90%.

## Authors' contributions

NC conceived this study, performed the analyses presented here and wrote the manuscript. IRS scientifically contributed to ideas presented here and contributed to writing the manuscript. Both authors read and approved the final manuscript.

## References

[B1] Palmgren MG, Axelsen KB (1998). Evolution of P-Type ATPases. Biochim Biophys Acta.

[B2] de Souza FSG, Gomes SL (1998). A P-type ATPase from the aquatic fungus *Blastocladiella emersonii *similar to animal Na, K-ATPases. Biochim Biophys Acta.

[B3] Catty P, d'Exaerd A, Goffeau A (1997). The complete inventory of the yeast *Saccharomyces cerevisiae *P-type transport ATPases. FEBS letters.

[B4] Wang S, Takeyasu KA (1997). Primary structure and evolution of the ATP-binding domains of the P- type ATPases in *Tetrahymena thermophila*. Am J of Physiol.

[B5] Shono M, Wada M, Hara Y, Fujii T (2001). Molecular cloning of Na+-ATPase cDNA from a marine alga *Heterosigma akashiwo*. Biochim Biophys Acta.

[B6] Benito B, Garciadeblas B, Schreier P, Rodriguez-Navarro A (2004). Novel P-type ATPases mediate high-affinity potassium or sodium uptake in fungi. Eukaryotic Cell.

[B7] Fietto LG, Pugliese L, Gomes SL (2002). Characterization and expression of two genes encoding putative Na, K-ATPase in the chytridiomycete *Blastocladiella emersonii*. Biochim Biophys Acta.

[B8] Hass H, Taylor TN, Remy W (1994). Fungi from the Lower Devonian Rhynie Chert – mycoparasitism. American Journal of Botany.

[B9] Heckman DS, Geiser DM, Eidell BR, Stauffer RL, Kardos NL, Hedges SB (2001). Molecular Evidence for the Early Colonization of Land by Fungi and Plants. Science.

[B10] Hedges SB, Blair JE, Venturi ML, Shoe JL (2004). A molecular timescale of eukaryote evolution and the rise of complex multicellular life. BMC Evolutionary Biology.

[B11] Remy W, Taylor TN, Hass H (1994). Early Devonian fungi – a blastocladalean fungus with sexual reproduction. American Journal of Botany.

[B12] Taylor TN, Remy W, Hass H (1994). Allomyces in the Devonian. Nature.

[B13] Schüßbler A, Schwarzott D, Walker C (2001). A new fungal phylum, the Glomeromycota: phylogeny and evolution. Mycol Res.

[B14] Jakobsen I, Varma A, Hock B (1995). Transport of phosphorus and carbon in VA mycorrhizas.

[B15] Newsham KK, Fitter AH, Watkinson AR (1995). Arbuscular mycorrhiza protect an annual grass from root pathogenic fungi in the field. J Ecol.

[B16] Van der Heijden MGA, Klironomos JN, Ursic M, Moutoglis P, Streitwolf-Engel R, Boller T, Wiemken A, Sanders IR (1998). Mycorrhizal fungal diversity determines plant diversity, ecosystem variability and productivity. Nature.

[B17] Redecker D, Kodner R, Graham LE (2000). Glomalean fungi from the Ordovician. Science.

[B18] Kuhn G, Hijri M, Sanders IR (2001). Evidence for the evolution of multiple genomes in arbuscular mycorrhizal fungi. Nature.

[B19] Hijri M, Sanders IR (2005). Low gene copy number shows that arbuscular mycorrhizal fungi inherit genetically different nuclei. Nature.

[B20] Corradi N, Kuhn G, Sanders IR (2004). Monophyly of β-tubulin and H^+^-ATPase gene variants in *Glomus intraradices*: consequences for molecular evolutionary studies of AM fungal genes. Fung Genet Biol.

[B21] Corradi N, Hijri M, Fumagalli L, Sanders IR (2004). Arbuscular mycorrhizal fungi (*Glomeromycota*) harbour ancient fungal tubulin genes that resemble those of the chytrids (*Chytridiomycota*). Fung Genet Biol.

[B22] Requena N, Breuninger M, Franken P, Ocon A (2003). Symbiotic status, phosphate, and sucrose regulate the expression of two plasma membrane H^+^-ATPase genes from the mycorrhizal fungus *Glomus mosseae*. Plant Physiol.

[B23] Koch AM, Kuhn G, Fontanillas P, Fumagalli L, Goudet J, Sanders IR (2004). High genetic variability and low local diversity in a population of arbuscular mycorrhizal fungi. Proc Natl Acad Sci USA.

[B24] Stukenbrock EH, Rosendahl S (2005). Clonal diversity and population genetic structure of arbuscular mycorrhizal fungi (*Glomus *spp.) studied by multilocus genotyping of single spores. Molecular Ecology.

[B25] Bruns TD, Vilgalys R, Barns SM, Gonzalez D, Hibbett DS, Lane DJ, Simon L, Stickel S, Szaro TM, Weisburg WG, Sogin ML (1992). Evolutionary relationships within the fungi: analyses of nuclear small subunit rRNA sequences. Mol Phyl Evol.

[B26] Forget L, Ustinova J, Wang Z, Huss VAR, Lang BF (2002). Hyaloraphidium curvatum: A linear mitochondrial genome, tRNA editing, and an evolutionary link to lower fungi. Mol Biol Evol.

[B27] Lutzoni F, Kauff F, Cox CJ, McLaughlin D, Celio G, Dentinger C, Padamsee M, Hibbett D, James TY, Baloch E, Grube M, Reeb V, Hofstetter V, Schoch C, Arnold AE, Miadlikowska J, Spatafora J, Johnson D, Hambleton S, Crockett M, Shoemaker R, Sung GH, Lücking R, Lumbsch T, O'Donnell K, Binder M, Diederich P, Ertz D, Gueidan C, Hansen K, Harris RC, Hosaka K, Lim YW, Matheny B, Nishida H, Pfister D, Rogers J, Rossman A, Schmitt I, Sipman H, Stone J, Sugiyama J, Yahr R, Vilgalys R (2004). Assembling the fungal tree of life: progress, classifications, and evolution of subcellular traits. American Journal of Botany.

[B28] Benito B, Garciadeblas B, Rodriguez-Navarro A (2002). Potassium- or sodium-efflux ATPase, a key enzyme in the evolution of fungi. Microbiology-SGM.

[B29] http://www.broad.mit.edu.

[B30] Martin F, Tuskan GA, DiFazio SP, Lammers P, Newcombe G, Podila GK (2004). Symbiotic sequencing for the Populus mesocosm. New Phytol.

[B31] Schwarzott D, Walker C, Schüssler A (2001). *Glomus*, the largest genus of arbuscular mycorrhizal fungi, is nonmonophyletic. Mol Phyl Evol.

[B32] Rokas A, Holland PWH (2000). Rare genomic changes as a tool for phylogenies. Trends Ecol Evol.

[B33] Keeling PJ, Palmer JD (2000). Parabasalian flagellates are ancient eukaryotes. Nature.

[B34] Sebat J, Lakshmi B, Troge J, Alexander J, Young J, Lundin P, Maner S, Massa H, Walker M, Chi M, Navin N, Lucito R, Healy J, Hicks J, Ye K, Reiner A, Gilliam TC, Trask B, Patterson N, Zetterberg A, Wigler M (2004). Large-scale copy number polymorphism in the human genome. Science.

[B35] Iafrate JA, Feuk L, Rivera MN, Listenwnik ML, Donahoe PK, Qi Y, Scherer SW, Lee C (2004). Detection of large scale variation in the human genome. Nat Genet.

[B36] Check E (2005). Human genome: Patchwork people. Nature.

[B37] Gonzalez E, Kulkarni H, Bolivar H, Mangano A, Sanchez R, Catano G, Nibbs RJ, Freedman BI, Quinones MP, Bamshad MJ, Murthy KK, Rovin BH, Bradley W, Clark RA, Anderson SA, O'Connell RJ, Agan BK, Ahuja SS, Bologna R, Sen L, Dolan MJ, Ahuja SK (2005). The Influence of CCL3L1 Gene-Containing Segmental Duplications on HIV-1/AIDS Susceptibility. Science.

[B38] Alkan N, Gadkar V, Coburn J, Yarden O, Kapulnik Y (2004). Quantification of the arbuscular mycorrhizal fungus Glomus intraradices in host tissue using real-time polymerase chain reaction. New Phytol.

[B39] St-Arnaud M, Hamel C, Vimard B, Caron B, Fortin JA (1996). Enhanced hyphal growth and spore production of the arbuscular mycorrhizal fungus *Glomus intraradices *in an in vitro system in the absence of host roots. Mycol Res.

[B40] Nagahashi G, Douds DD (1999). Rapid and sensitive bioassay to study signals between root exudates and arbuscular mycorrhizal fungi. Biotech Tech.

[B41] Altschul SF, Gish W, Miller W, Myers EW, Lipman DJ (1990). Basic local alignment search tool. J Mol Biol.

[B42] Thompson JD, Higgins DJ, Gibson TJ (1994). Improving the sensivity of progressive multiple sequence alignment through sequence weighting, position specific gap penalties and matrix choice. Nucleic Acids Res.

[B43] Kumar S, Tamura K, Jakobsen IB, Nei M (2002). MEGA2: molecular evolutionary genetics analysis software. Bioinformatics.

[B44] Kumar S (1996). Stepwise algorithm for finding minimum evolution trees. Mol Biol Evol.

